# *Alternaria* toxins alternariol and alternariol monomethyl ether in grain foods in Canada

**DOI:** 10.1007/s12550-012-0141-z

**Published:** 2012-09-04

**Authors:** Peter M. Scott, Wendy Zhao, Sherry Feng, Benjamin P.-Y. Lau

**Affiliations:** Health Canada, Food Research Division, 251 Sir Frederick Banting Driveway, Ottawa, ON K1A 0K9 Canada

**Keywords:** Alternariol, Alternariol monomethyl ether, Liquid chromatography, Tandem mass spectrometry, Grain foods

## Abstract

*Alternaria alternata* has been reported to be the most common fungus on Canadian Western wheat. The *Alternaria* toxins alternariol (AOH) and alternariol monomethyl ether (AME) are mutagenic in vitro and there is also limited evidence for carcinogenic properties. They have been found in wheat from Europe, Argentina, China and Australia, but they have not been looked for in Canadian grains or grain foods. In the present study, 83 samples of grain-based food sold in Canada, including flour, bran, breakfast cereals, infant cereals and bread, were analysed for AOH and AME using extraction with methanol, clean-up on combined aminopropyl/C18 solid phase extraction (SPE) columns, and liquid chromatography (LC) with tandem mass spectrometric (MS/MS) determination. The overall average recoveries of AOH and AME from a variety of spiked cereal foods (*n* = 13) were 45 ± 9 % and 53 ± 9 %, which could be attributed mainly to MS matrix effects The instrumental limits of detection (LOD) were 0.34 ng/g and 0.13 ng/g for AOH and AME, respectively, and the instrumental limits of quantitation (LOQ) were 1.1 and 0.43 ng/g. Of 83 samples analysed, 70 were positive for AOH (up to 63 ng/g, in a soft wheat bran) and 64 contained AME (up to 12 ng/g in a bran-based breakfast cereal). Of particular interest was the presence of AOH and/or AME in 27 out of 30 infant foods (up to 4.4 ng/g and 9.0 ng/g, respectively, in a sample of multigrain cereal).

## Introduction

Alternariol (AOH) and alternariol monomethyl ether (AME) are the main benzopyrone mycotoxins produced by *Alternaria alternata*. They were first isolated and structurally characterized as 3,7,9-trihydroxy-1-methyl-6*H*-dibenzo[*b,d*]pyran-6-one and 3,7-dihydroxy-9-methoxy-1-methyl-6*H*-dibenzo[*b,d*]pyran-6-one, respectively, 60 years ago (Raistrick et al. 1953). Other species of *Alternaria* (Ostry 2008; Logrieco et al. 2009), *Stagonospora nodorum* (Tan et al. 2009) and *Phomopsis* strains (Abreu et al. 2012) have also been found to produce AOH and AME. The toxicological database on AOH and AME is limited. Although their acute toxicity in animals is low, they are mutagenic in vitro and there is also some evidence for carcinogenic properties in unconventional assays (Brugger et al. 2006; Ostry 2008; EFSA 2011): pre-cancerous changes were observed in the oesophageal mucosa of mice fed AME for 10 months (Yekeler et al. 2001); human embryo tissue treated with AOH caused subcutaneous induction of squamous cell carcinoma in mice (Liu et al. 1992); NIH/3T3 cells transformed by AME caused tumours subcutaneously in mice (Liu et al. 1991); and AOH and AME induced DNA strand breaks in cell cultures (Fehr et al. 2009).

Natural occurrences of AOH, AME, and in some cases other *Alternaria* toxins have been reported in various fruits, processed fruit products such as apple juice, tomato products, wheat and other grains, sunflower seeds, oilseed rape meal, flax seed, linseed and pecans (Ostry 2008; Logrieco et al. 2009). They have also been found in Canadian lentils (Ostry et al. 2004). Another *Alternaria* toxin, tenuazonic acid, was recently found in beer and other cereal foods (Siegel et al. 2010b; Asam et al. 2012). Currently, there are no regulations anywhere in the world for the presence of *Alternaria* toxins in food or feed.

AOH and AME have been found in wheat from Europe, Russia, Kenya, Argentina, China and Australia (Gruber-Schley and Thalmann 1988; Grabarkiewicz-Szczesna and Chelkowski 1993; Webley et al. 1997; Li and Yoshizawa 2000; Scott 2001; Müller et al. 2002; Azcarate et al. 2008; Ostry 2008; Logrieco et al. 2009; Burkin and Kononenko 2011; Wagacha et al. 2010). *Alternaria alternata* has been reported to be the most frequently isolated fungus from western Canadian wheat (Clear et al. 2005), but *Alternaria* toxins have not been looked for previously in Canadian grains or grain-based foods. AOH and AME were stable under wet baking conditions as in bread baking (Siegel et al. 2010a) so they might be expected to occur in Canadian grain-based foods. In fact the incidence of these mycotoxins in grain-based foods was low in European studies (Asam et al. 2011; EFSA 2011). The present report describes the use of liquid chromatography (LC)-tandem mass spectrometry (MS/MS) to survey Canadian grain-based foods for AOH and AME.

## Materials and methods

Most samples were obtained from retail stores in Canada. They were ground if necessary, then each sample (2.5 g) was homogenized with 25 ml extraction solvent (methanol), followed by centrifuging. Ten millilitres of water was added to 1 ml extract and the mixture loaded onto a combined aminopropyl/C18 solid phase extraction (SPE) column (Sorbent Technologies, Atlanta, GA) previously conditioned with 5 ml methanol and 5 ml water. The column was washed with 3 ml 35 % acetonitrile and 3 ml water. Toxins were eluted with 3 ml acetonitrile-acetic acid (100:1, v/v). The eluate was evaporated to dryness at 40–45 °C under nitrogen and the residue was dissolved in 500 μl methanol (some residues from negative samples of wheat flour and wheat bran were dissolved in 200 μl), then filtered.

LC-MS/MS was performed on a Waters Acquity UPLC with a Phenomenex (Torrance, CA, USA) Gemini-NX C18, 2.0 × 150-mm, 3-μm column at room temperature coupled to a Waters Quattro-Premier XE Triple Quadrupole mass spectrometer (Milford, MA, USA). Autosampler tray temperature was 5 °C. The flow rate was 0.175 ml/min. Injection volume was 10 μl. Mobile phases were: *A* = 100 % H_2_O and *B* = 100 % methanol with the following gradient: 30 % B up to 6.0 min, 80 % B from 6.0 to 17.0 min, then 30 % B.

Alternatively, depending on instrument availability, LC-MS/MS was carried out on an Agilent 1,200 liquid chromatograph coupled to a Micromass Quattro Ultima Triple Quadrupole MS/MS, using a Jones (Grace Davison, Discovery Sciences, USA) Genesis C18, 2.1 × 150-mm, 3-μm column at 30 °C with a gradient system of 30–80 % methanol in water similar to that above except staying at 80 % methanol up to 18 min.

**Fig. 1:**
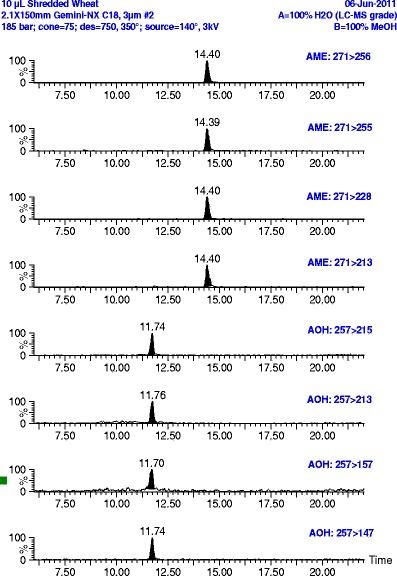
LC-MS/MS analysis (using the Micromass Quattro Ultima) of AME (9.0 ng/g) and AOH (11 ng/g) in a 100 % whole grain shredded wheat sample

Negative ion electrospray ionization MS/MS conditions included capillary voltage: −3.0 kV, cone voltage 20 V (on the Ultima instrument) or 40 V (on the Premier instrument), source temperature 120 °C (Premier) or 140 °C (Ultima), desolvation temperature was 380 °C (Premier) or 350 °C (Ultima), cone gas (N_2_) flow 50 l/h, desolvation gas (N_2_) flow 600 l/h, collision gas (Ar) pressure 3.10 × 10^−3^ mbar, multiplier voltage 625 V (Ultima) or 650 V (Premier). Multiple reaction monitoring (MRM) analysis (dwell time 0.080 s) had four transitions per compound (see Fig. 1). Collision energies for each MRM transition are given in Table 1. Results for three transitions were averaged for the food analyses (Tables 2, 3, 4, 5 and 6).

**Table 1:** Collision energies for AOH and AME MRM transitions on two instruments

MRM transition	Collision energy (Premier), eV	Collision energy (Ultima), eV
257 → 147	32	32
257 → 157	30	25
257 → 213	23	25
257 → 215	25	25
271 → 213	35	37
271 → 228	30	27
271 → 255	32	30
271 → 256	22	20

**Table 2:** AOH and AME in flour and bran

Sample	AOH (ng/g)	AME (ng/g)
Whole wheat flour	0.5	0.5
Hard wheat flour 1	nd	nd
Hard wheat flour 2	nd	nd
Hard wheat flour 3	nd	nd
Soft wheat flour 1	0.5	nd
Soft wheat flour 2	nd	nd
Durum wheat flour	nd	nd
Durum wheat bran 1	nd	nd
Durum wheat bran 2	nd	nd
Durum wheat bran 3	nd	nd
Hard wheat bran 1	6.0	1.0
Hard wheat bran 2	2.1	2.5
Soft wheat bran 1	3.0	2.5
Soft wheat bran 2	63	8.9
Soft wheat bran 3	nd	nd

*nd* less than LOD

**Table 3:** AOH and AME in breakfast cereals

Sample	AOH (ng/g)	AME (ng/g)
Oats cereal	1.0	1.0
Oatmeal cereal	0.8	0.8
Wheat cereal	2.5	3.0
Ring-shaped cereal	0.4	0.4
Mixed cereal 1	0.6	0.4
Mixed cereal 2	0.6	0.4
Shredded wheat cereal 1	6.0	3.5
Shredded wheat cereal 2	7.0	3.0
100 % Whole grain shredded wheat cereal	11	9.0
100 % Bran cereal	35	12

**Table 4:** AOH and AME in bread

Sample	AOH (ng/g)	AME (ng/g)
Sovital bread	1.5	0.5
White bread 1	0.4	nd
White bread 2	0.5	nd
White bread 3	0.6	nd
White bread 4	0.6	nd
White bread 5	0.8	0.3
White bread 6	1.1	0.5
60 % Whole wheat bread	5.0	2.1
100 % Whole grain wheat bread 1	2.6	1.3
100 % Whole grain wheat bread 2	3.0	1.0
100 % Whole wheat granola bread	1.0	nd
100 % Whole wheat bread 1	0.7	nd
100 % Whole wheat bread 2	5.3	1.4
100 % Whole wheat bread 3	2.0	0.6
100 % Whole wheat bread 4	1.0	0.2
100 % Whole wheat bread 5	1.9	0.6
100 % Whole wheat bread 6	2.1	0.8
100 % Whole wheat bread 8	0.8	nd
Bran bread	2.2	0.4
Rye bread 1	6.7	3.0
Rye bread 2	1.4	0.6
Rye bread 3	1.3	0.4
Rye bread 4	0.9	0.2
Rye bread 5	1.2	0.3
Multigrain bread 1	0.8	0.3
Multigrain bread 2	1.6	0.6
Multigrain bread 3	3.3	0.8
Multigrain bread 4	0.6	0.2
Cinnamon raisin bread	1.1	0.3

*nd* less than LOD

**Table 5:** AOH and AME in infant foods

Sample	AOH (ng/g)	AME (ng/g)
Biscuits 1	1.1	nd
Biscuits 2	1.2	1.0
Teething biscuits 1	nd	0.9
Teething biscuits 2	1.0	0.9
Apple biscuits	0.7	0.7
Toddler biscuits	0.6	0.7
Cookies and biscuits	nd	1.1
Oatmeal cookies and biscuits	0.7	0.8
Graham cookies 1	0.8	0.7
Graham cookies 2	1.3	0.9
Graham cookies 3	1.4	0.9
Crackers 1	1.1	0.9
Crackers 2	1.2	0.9
Graham crackers	nd	0.7
Wheat cereal	0.6	0.5
Wheat & oat cereal	0.6	0.7
Wheat, honey & flakes	0.7	0.7
Wheat biscuit cereal	0.5	0.5
Wheat, yogurt & blueberry cereal	0.8	0.7
Wheat with mixed fruit cereal	0.6	0.7
Wheat and rice cereal	0.5	0.6
Rice cereal	0.7	0.8
Mixed grain with fruit cereal	0.7	0.7
Mixed cereal 1	0.7	0.9
Mixed cereal 2	1.4	0.8
Mixed cereal with fruits	0.6	0.7
Multigrain cereal 1	4.4	9.0
Mixed grain cereal	1.9	2.0
Organic barley cereal	0.7	0.8

*nd* less than LOD

**Table 6:** Summary of AOH and AME in grain foods

Sample group	n	AOH positive	AOH range (ng/g)	AME positive	AME range (ng/g)
Flour, bran	15	6	nd−63	5	nd-8.9
Breakfast cereal	10	10	0.4–35	10	0.4–12
Bread	29	29	0.4–6.7	22	nd-3.0
Infant food	29	25	nd−4.4	27	nd-9.0

*nd* less than LOD

The instrumental limits of detection (LOD) (based on S/N ratio = 3) for AOH and AME were determined using the weakest response among the three MRM transitions for AOH (m/z 257 → 215; m/z 257 → 213; m/z 257 → 147) and for AME (m/z 271 → 256; m/z 271 → 255; m/z 271 → 228). A positive result implies the presence of positive responses from all three MRM transitions at the same correct retention time of the analyte. The instrumental limits of quantitation (LOQ) were defined as 3.3-times the instrumental LOD. The overall method LOQ would be higher if extraction and clean-up recoveries and matrix effect were to be factored in. To determine matrix effects, AOH and AME standards equivalent to 20 ng/g grain food were added in 50 % acetonitrile to extracts of whole wheat flour and four other grain foods before LC-MS/MS injection. The matrix effect was then calculated as the ratio (expressed in percentage) between the response of the analyte in the spiked sample to that of the same amount of standard in solvent, after the correction for the background level in the blank matrix (if present).

Overall method recoveries were determined in triplicate by spiking samples of grain food in which AOH and AME were not detected or if they were, their concentrations were subtracted from those determined in the spiked samples.

## Results and discussion

As LC-UV was insufficiently sensitive at low ng/g levels, LC-MS/MS was used for quantitation of AOH and AME in extracts of the 83 cereal foods analysed. The limits of detection (LOD) were 0.34 ng/g and 0.13 ng/g for AOH and AME, respectively, and the limits of quantitation (LOQ), defined as 3.3-times the LOD, were 1.1 and 0.43 ng/g, respectively. The instrumental LODs obtained from the Ultima LC-MS/MS system (0.33 ng/g) for AOH and 0.13 ng/g for AME) were almost identical to those obtained from the Quattro-Premier LC-MS/MS system (0.34 ng/g for AOH and 0.13 ng/g for AME). For this reason, a single value of LOD was used for each analyte.

LC-MS/MS has been used in two previous publications for determination of AOH and AME in grain foods (Siegel et al. 2010a; Asam et al. 2011). We found overall method recoveries of AOH and AME from 13 foods averaged 45 and 53 %, respectively, ranging from 32 % (bran bread) to 58 % (wheat and barley infant cereals) for AOH and 37 % (bran bread) to 71 % (rice cereal) for AME (Table 7). LC-MS/MS matrix effects were determined for five foods. Signals of standard AOH and AME in the whole wheat flour extract averaged 59 % and 53 %, respectively, compared with the standards in 50 % acetonitrile (Table 8); for hard wheat bran the signal suppression averaged 40 % and 50 %. Extracts of oatmeal cereal, whole wheat bread and white bread showed signal enhancement for AME. The overall method recoveries could be attributed to matrix effects (Table 8) in two cases where comparisons with overall method recoveries were made (whole wheat flour and hard wheat bran). Unfortunately, [^13^C]-labelled AOH and AME were not available for use as internal standards in a stable isotope dilution assay (SIDA).

**Table 7:** Recoveries of AOH and AME from different cereal food matrices (± standard deviation)

Matrix	*n*	Added level (ng/g)	AOH (%)	AME (%)
Whole-wheat flour	3	10	35 ± 6	45 ± 10
Wheat breakfast cereal	3	10	40 ± 2	50 ± 5
Hard wheat bran	3	10	47 ± 14	52 ± 8
Rice cereal	3	10	49 ± 3	71 ± 10
Wheat cereal	3	10	58 ± 2	65 ± 0.3
Biscuits	3	10	56 ± 3	40 ± 1
Organic barley baby cereal	3	10	58 ± 2	53 ± 4
60 % Whole-wheat bread	3	10	34 ± 4	54 ± 10
White bread	3	10	38 ± 8	59 ± 14
100 % Whole-grain-wheat bread	3	10	44 ± 9	49 ± 9
Bran bread	3	10	32 ± 6	37 ± 6
Rye bread	3	10	49 ± 5	54 ± 9
Cinnamon raisin bread	3	10	45 ± 5	56 ± 5
Average			45 ± 9	53 ± 9

**Table 8:** LC-MS/MS matrix effects

Extract	AOH recovery (%)	AME recovery (%)
Blank	100	100
Whole-wheat flour (*n* = 3)	59	53
Oatmeal cereal (*n* = 2)	67	104
Whole-wheat bread (*n* = 1)	84	110
White bread (*n* = 2)	88	98
Hard wheat bran (*n* = 2)	40	50

AOH and AME were found in a wide range of grain foods grouped into flour and bran, breakfast cereals, bread, and infant foods (Tables 2, 3, 4, 5 and 6). A reliable comparison between food types is not possible considering the small number of samples in most cases (e.g. only one rice cereal and one barley cereal were analysed in the infant foods group). Concentrations of AOH and AME found in the grain foods were not corrected for the overall average method recoveries as an *F*-test showed that recovery variances for 13 different matrices (Table 7) were not uniform. It is noteworthy that AOH and/or AME were detected in 25 out of 29 infant foods (up to 4.4 ng/g and 9.0 ng/g, respectively, in a sample of multigrain cereal) (Table 5).

Compared with surveys on grains themselves, there are few other surveys in the literature for AOH and/or AME in grain foods (Siegel et al. 2010a; Asam et al. 2011; EFSA 2011). In the EFSA’s recent scientific opinion on *Alternaria* toxins in feed and food, maximum concentrations of AOH and AME in the *Grains and grain-based products* food category were reported to be 256 ng/g and 86 ng/g, respectively (EFSA 2011). Mean concentrations for lower and upper bound estimates ranged from 1.8 to 7.3 ng/g for AOH and from 0.37 to 1.99 ng/g for AME. The EFSA noted that concentrations in grain milling products were generally lower in comparison with the grains themselves. Levels and incidences were extremely low and no toxins were detected in 49 samples of foods for infants and small children (the type was not specified) (EFSA 2011). However, the EFSA detection limits (for all foods) varied from 0.01 to 6.0 ng/g for both AOH and AME, with the median detection limit for each being 6.0 ng/g and 1.0 ng/g, respectively (EFSA 2011). Siegel et al. (2010a) found < 15 ng/g of AME in a sample of buckwheat cookies (we did not analyse any buckwheat products) and Asam et al. (2011) detected 4.1 ng AOH/g in a sample of spelt flour and AME in two samples of oat flakes for human consumption (< 1 ng/g); levels of AOH and AME in our single samples of oats cereal and oatmeal cereal were also very low (Table 3).
